# Looking at Infertility Treatment through The Lens of The Meaning of Life: The Effect of Group Logotherapy on Psychological Distress in Infertile Women

**Published:** 2013-03-03

**Authors:** Leili Mosalanejad, Anahita Khodabakshi Koolee

**Affiliations:** 1 Department of Mental Health, Jahrom University of Medical Sciences, Jahrom, Iran; 2 Department of Family Counseling, University of Social Welfare and Rehabilitation Science, Tehran, Iran

**Keywords:** Spiritual Therapies, Psychological Distress, Mental Health, Infertility

## Abstract

**Background:**

Women in particular suffer from psychological stress when diagnosed with infertility. Psychosocial interventions are known to not only prevent and lessen various mental problems, but also to play a positive role in physical health and pregnancy rates. The aim of this study is to determine the unique impact of spiritual psychotherapy on concerns about infertility and their perceived psychological stresses.

**Materials and Methods:**

This study was a randomized clinical trial. The study population included nearly 800 infertile couples who attended the Maternity and Gynecology Clinic of Jahrom University of Medical Sciences, Jahrom, Iran. We enrolled65 people who were randomly divided into two groups, experimental (n=33)and control (n=32). The experimental group received spiritual group psychotherapy counseling for 12 sessions, 2 hours per week for a 3 months period. The control group did not receive any intervention, but due to ethical considerations, we gave a presentation (one session) about infertility treatment for this group after the research process was completed. We used two questionnaires to obtain data, the Penn State Worry Questionnaire (PSWQ) and Perceived Stress Scale (PSS). Data analysis was done by descriptive and analytic statistics using SPSS 16 software.

**Results:**

Psychological intervention in the treatment group significantly decreased the PSWQ (p=0.004). There were significant differences in the mean score of the PSWQ in both groups as determined by analysis of covariance (ANCOVA; p=0.009). Psychological intervention in the treatment group decreased the level of perceived stress, when compared with the control group. According to ANCOVA there were significant differences between the mean PSS scores of both groups (p=0.01).

**Conclusion:**

Logotherapy is related to stress reduction and can decrease psychiatric symptoms of worry and perceived stress. This approach tends to improve an infertile person's ability to deal with their problem of finding the meaning of life. Thus it can be concluded that logotherapy along with other treatment methods, is a useful approach for infertile couples (Registration Number:IRCT201108247407N2).

## Introduction

Approximately 10 to 15% of child bearing age couples experience infertility. Infertility is a stressful event that can give rise to psychological difficulties. This problem affects multiple areas of life, including physical, emotional, financial, social, and psychological (1). A common theme in explaining the stressful impact of infertility is that it represents a life-crisis, and perhaps due to this reason people experience grief reactions similar to those experienced with bereavement (2, 3).

Wischmann explained that women who have experienced infertility face psychological disturbances, including low self-esteem and other mental health problems. For this reason counseling and psychological intervention could help them achieve a healthy, high quality of life (4).

Evidence suggests that the anxiety and depression associated with infertility are similar to those associated with other serious medical conditions such as cancers and infection with human immunodeficiency virus (5). Other studies have stressed that psychosocial elements, including dysfunctional coping strategies for stress, anxiety and depression may possibly lower one’s chances of becoming pregnan (6-8).

Currently, the number of clients who seek infertility treatments is increasing because of women’s interests in delaying pregnancy and their increased awareness about infertility treatment. Couples who are aware of the effects of psychosocial treatment on increasing the chances of pregnancy are quite motivated to participate in counseling and psychotherapy sessions (9).

Psychosocial interventions are known to not only prevent and lessen various mental problems such as anxiety, depression, and phobias, but also to play a positive role in physical health and achieving pregnancy (10). Psychosocial counseling as an intervention strategy has a dramatically effect when given prior to the onset of infertility treatment. These interventions could increase couples outlooks and guide them to succeed in achieving pregnancies (11) .

Humans are more susceptible to a variety of illnesses when they suffer from feelings of meaninglessness or existential frustration. ‘‘It has been considered that some form of the meaning of life does exist for individuals, although it has to be discovered, and that the meaning of life for an individual can neither be given nor created, it has to be found or discovered’’ (12). Infertility can create a sense of meaninglessness and lack of feeling in spouses. Numerous psychological and counseling methods and theories that could be applicable for infertility exist, however one of the most influential is logotherapy.

Logotherapy is an existential psychotherapy that can be used with patients who have psychological and mental disorders. The center of logotherapy is self-transcendence, a pathway that increases the sense of purpose, which in turn enhances the sense of well-being and the ability to cope with suffering and stress. This approach is based on the assumption that fulfillment in life is the best protection people from emotional instability (13).

Logotherapy assists those who suffer from mental disorders, neuroticism, or psychopathic problems and enables individuals to break through their high expectations and disappointments (14).

Frankl created logotherapy, which came from his life experiences. He has stated that human beings search for the meaning of life and they are motivated to discover and elaborate on that meaning. According to Frankl human existence has three components: soma, psychological, and spiritual. These aspects comprise the “self” and they are related to each other. Frankl suggested that" the most difficult psychological issue facing modern people is existential emptiness due to a lack of meaning in life, and developed logotherapy to overcome this most challenging hurdle" (15).

Meaning in life is described as an important goal that adds purpose to life. It is a powerful forcein humans and is taken into consideration in logotherapy treatments. Logotherapy includes the following techniques of paradoxical intention: dereflection, hyper-reflection, attitude modification, Socrates talks, and self-transcendence (13).

According to Farankel the process of analyzing the meaning of life follows a few fundamental philosophical elements that include: i. life has meaning, ii. We derive motivation from this meaning, iii. we have freedom to search for our own meaning and iv. the components of human being self are-consists of soma(physical), psyche, and truth (16).

A human being does not consist of just somatic health and environmental forces, but is free to take a stand toward inner conditions and outer circumstances (16). Health services could be considered as possible sources of the relationship between mental and physical health. Kang and Kim developed a logotherapy intervention and investigated how this program affected patients with end-stage cancer in terms of their suffering, meaning in life, and spiritual well-being. This study has proposed that logotherapy can be a useful program for patients with terminal cancerin addition to decreasing distress and improving quality of life (17). Many studies place emphasis on logotherapy as an important approach to chronic diseases. One of the studies has researched the effect of this approach on adolescents with cancerand identified emotional care as a necessity for optimizing the care for young cancer patients (18, 19). Infertile people suffer greatly due to their condition. “People who faced a problem of one or more in normal or usual physiological, psychological, social, and/or spiritual functioning they might try to finding the meaning among their suffers’’(20). No previous study has examined the effectiveness of logotherapy for infertile individuals.The aim of this study is the unique impact of group logotherapy on the concerns of infertility and its perceived stress, defined as both suffering and psychological stress.

## Materials and Methods

This study was a randomized clinical trial that included all infertile couples who attended the Maternity and Gynecological Clinic at Jahrom University of Medical Sciences, Jahrom, Iran. Infertility was defined as at least one year of unprotected coitus without conception. Among 800 women or couples, 80 met the following inclusion criteria: history of primary infertility, no somatic or psychiatric problems, residents of Jahrom, between 20-40 years of age, had a personal mobile phone, were literate, and expressed interest in participating in regular group meetings. At the onset of the study, we enrolled the 80 females who met the inclusion criteria.

Study participants were randomly divided into two groups (experimental and control) according to their file numbers. During the first two sessions, 15 people were absent or did not complete the questionnaire in its entirety and therefore they were excluded. The duration of the study was conducted with 65 participants, 33 in the experimental group and 32 in the control group who regularly attended the meetings after the end of study. Participants in the experimental group received group logo therapy for 12 weekly sessions. All participants elaborated on their own positive meaningful messages in the sessions, and a brief summary of each session was sent to each person at the end of every two sessions in order to achieve maximum performance. The control group did not receive any intervention, but due to ethical considerations, we gave a presentation (one session) about infertility treatment for this group after the research process was completed. Participant consent was acquired and the research project was approved by the Ethics Committee at Jahrom University of Medical Sciences.

The logotherapy educational program is an intervention program. The executive package has been used by various researchers in both chronic and severe diseases (20). The main objective of the sessions according to previous research was considered and then adjusted to meet the objectives of the current study.

### Objectives of the sessions

The objectives of the sessions are as follows:

1. Identify the goals and rules of the meeting, the familiarity of members with each other, and the expression of the meaning of life, which are steps that aim to create trust within the group and promote group dynamics (1 session).
2. Gather information from patients' characteristics and capabilities as the center of self-consciousness and reflection into anxiety-related factors and determining all strategies to expose anxiety (2 sessions).
3. Necessity of maintaining one's personal identity and how to interact with others as a way to find the meaning of love (1 session).
4. How to establish good relations with families and use different approaches to counseling date families so that they can search for the meaning of life through strengthening family ties (1 session).
5. The meaning of suffering, finding the hidden meaning of the infertility problem by emphasizing the philosophy of life and marriage (2 sessions).
6. Identifying assisted reproductive therapies and providing information about all treatment options as a way to creating hope (2 sessions).
7. Recognizing the value of creating the kind of work and service to others who can give meaning to life (i.e., helping charities, organ donation, etc.) (1 session).
8. Understanding the empirical values of the meaning of life and its value in addressing nature, the deepening of life through interaction with nature, and pursuing art. (Seeing the beauty that exists in nature and art to coping with -the - challenges) (1 session).

In one session, we have included trend values that discuss situations where people are powerless to deal with and are forced to accept that situation (i.e., living without a child).

Data were gathered from two questionnaires, the PSWQ and the PSS. The PSWQ investigates clinical and non-clinical groups of adults and consists of 16Likert items. This questionnaire has excellent internal consistency, test-retest reliability, and concurrent and discriminative validities. In a clinical group study by Brown et al. (21), the PSWQ displayed high reliability and validity. The PSWQ has been previously normalized to the Iranian society (22, 23).

The PSS is a 14 item self-report questionnaire that measures a person's evaluation of the stressfulness of situations during the past month of their lives. This questionnaire is the most widely used psychological tool for measuring the perception of stress.‘‘It is a measure of the degree to which situations in one’s life are appraised as stressful. Items were designed to tap how unpredictable, uncontrollable, and overloaded respondents find their lives. The scale also includes a number of direct queries about current levels of experienced stress’’. Subjects responses are measured on a five-point scale, 0 (never), 1 (almost never), 2 (sometimes), 3 (fairly often), and 4 (very often). This self-report test is from Cohen et al. (24), who have established its reliability and validity (r=0.85) and internal reliability in American (r=0.60) and Iranian (r=0.81) populations. These tests have been used by numerous Iranian researchers, and have been normalized in the Iranian community (25, 26). We used descriptive analysis as a frequency and percentage of data distribution; analytic statistics was used as the paired t-test and student t test compared the mean of data within a group and between groups. Analysis of covariance (ANCOVA) compared the significant mean of data in the two groups.

## Results

The current study included 33 infertile women in the treatment group and 32 infertile women in the control group. The demographic characteristics of the two groups did not significantly differ in terms of age (p=0.43), education (p=0.13), duration of infertility, and etiology of infertility (p=0.26).

In the two groups, 44.6% of the women were between the ages of 20-30 years and 49.2% were between the ages of 31-40 years. Education level was similar in the treatment and control groups (p=0.13).There were 59.5% of participants in the treatment group and 59.5% of control group participants who had high school educations. Infertility duration in the treatment group was 12 (36.5%) years and in the control group, it was 16(51.6%) years. There were no significant differences between the two groups (p=1).

Female factor infertility was observed in 18 (56.2%) of participants in the treatment group and 16 (51.6%) in the control group. Male factor infertility was observed in 3 (9.4%) participants in the treatment group and 7 (22.6%) in the control group. The cause of infertility was similar in both groups (p=0.26; Table 1).

**Table 1:** Demographic characteristics of the treatment and control groups


Variable	Experimental group (n%)	Control group (n%)	P value

**Age (Y)**			
**20-30**	15 (23)	14 (21.6)	1.71 (0.43)
**31-40**	15 (23)	17 (26.2)	
**>40**	2 (3.1)	2 (3.1)	
**Education**			
**Primary**	6 (15.5)	11 (32.3)	-1.52 (0.13)
**Middle-high School**	19 (59.5)	18 (58.1)	
**Diploma**	7 (21.7)	1 (3.2)	
**University degree and above**	1 (3.3)	2 (6.5)	
**Duration of infertility (Y)**			
**1-3**	12 (37.5)	9 (29)	-1.11 (0.26)
**4-6**	5 (15.6)	6 (19.4)	
**7-10**	11 (34.4)	16 (51.6)	
**>10**	4 (12.5)	0 (0)	
**Etiology of infertiltiy**			
**Female factor**	18 (56.2)	16 (51.6)	-1.11 (0.26)
**Male factor**	3 (9.4)	7 (22.6)	
**Unknown**	11 (34.4)	8 (25.8)	


Psychological intervention in the treatment group caused a significant decrease in the PSWQ score from 33.25 ± 12.24 to 27.31 ± 13.50 (p=0.004). The stress score in the control group was 34.19 ±8.80 before and 34.45 ± 8.23 after the study, which was not significant (p=0.65; Table 2).

**Table 2:** Differences between mean score of Penn State Worry Questionnaire between groups, pre and post-test


Group	Pre-test Mean (SD)	Post-tes Mean (SD)	t-student	P value

**Experimental**	33.25 (12.24)	27.31 (13.50)	3.06	0.004*
**Control**	34.19 (8.80)	34.45 (8.23)	-0.45	0.65


*; P<0.05 is significant.

**Table 3:** Differences between mean score of Penn State Worry Questionnaire (PSWQ) in the two groups with ANCOVA


Group	Mean (SD)	F-test	P value

**Pre-test Experimental**		5.21	0.27
	33.25(12.24)		
**Control**	34.19(8.80)		
**Post-test Experimental**		11.37*	7.28*
	27.31(13.50)		
**Control**	34.45(8.23)		


*; P≤0.001 is significant.

Other results showed no significant differences between the mean PSWQ scores. Rather, differences in the mean scores of the PSWQ in the two groups were significant after intervention (p=0.01), as confirmed by ANCOVA (p=0.009; Table 3).

**Table 4:** Differences in mean score of the Perceived Stress Scale (PSS) between the two groups, pre and post-test with ANCOVA


Group	Pre-test Mean (SD)	Post-test Mean (SD)	F-test	P value

**Experimental**	29.25(4.75)	28.18(4.94)	1.11	0.27
**Control**	29.09(4.79)	28.29(4.62)	1.12	0.27


Results of the PSS are presented in table 4. Psychological intervention in the treatment group decreased the level of stress (29.25 ± 4.75 vs. 28.18 ± 4.94; p=0.27). In the control group there was also a decreased level of stress (29.09 ± 4.79 vs. 28.29 ± 4.62; p=0.27), however neither of the scores were significant (Table 4).

There were significant differences between mean scores of the PSS after intervention in mean stress in the two groups by ANCOVA, as a level of total perceived stress (F=7.05, p=0.01) in the treatment group decreased more than in the control group (Table 5).

**Table 5:** Differences between mean scores of the PSS between groups according to ANCOVA


	Experimental Mean (SD)	Control Mean (SD)	F-test	P value

**Pre-test**				
**Perceived stress**	29.25 (4.75)	29.09 (4.79)	0.31	0.18
**Positive PSS**	17.93 (3.5)	16.48 (3.27)	0.40	0.24
**Negative PSS**	14.43 (3.50)	15.51 (5.07)	2.82	2.39
**Post-test**				
**Perceived stress**	28.18 (4.94)	28.29 (4.62)	0.33	7.05*
**Positive PSS**	17.37 (4.64)	15.58 (2.99)	5.1	3.65*
**Negative PSS**	14.90 (3.68)	15.22 (4.31)	0.46	1.30


*; P≤0.05 is significant.

## Discussion

According to our results the experimental group reported decreased worry and stress perception. The relationship between logotherapy and medicine has been the focus of considerable interest in recent years. Studies have suggested that many patients believe spirituality plays an important role in their lives, that there is a positive correlation between one's spirituality and health outcomes, and that patients would like physicians to consider these factors in their medical care (27).

Logotherapy is an educational program to activate comprehensive human critical power. This process stimulates and activates human brain function (28). One study has described the use of logotherapy (healing through meaning) for the treatment of combat-related post-traumatic stress disorder (PTSD). This study showed that logotherapy offers the combat veteran who struggled with existential issues hope for the "meaning of life". This study has also emphasized that, when veterans express higher levels of fulfillment, they were more accepting of stress and life events, and less deterred by their symptoms (29).

Recent results have also shown that this approach decreases worry from infertility symptoms. Others have studied the impact of this approach on treatment of serious diseases. Logotherapy, according to one study, was an effective approach that supported adolescent cancer patients to find meaning in their lives and successfully reduced their suffering. As spiritual intervention in a medical center, logotherapy has demonstrated effective promotion of the quality of life and prevented hopelessness caused by illness for patients under somatic stressful events (17). Research has confirmed the psychological impacts of logotherapy on chronic diseases. Another study on non-clinical patients by Lee has shown that the experimental group had a significant difference in their meaning of life and ego integrity compared to the control group. Therefore, logotherapy can be recommended as an effective approach for adolescents, adults and the ageing population (29). According to research, clinicians can facilitate healing by helping their patients find meaning in their illness (30).

It has been argued by Koenig et al. that meaning of life is purely a measure of emotional well-being (31). Finding the meaning in life is strongly correlated with functional well-being (32).

‘‘Spirituality intervention plays in the patient’s ability to cope with the illness. Although the spirituality may be a source of support but in some cases may be a source of emotional turmoil and stress’’(33). In one of study, 90% of women with spontaneous premature ovarian failure reported that spiritual intervention played an important role in helping them adjust to the emotional response to the infertility diagnosis and invasive procedures (34). Numerous evidence support the positive impact of logotherapy on patients’ psychological symptoms.

One study of group logotherapy on life expectation in cancer patients has shown that this therapy increased life expectation (35). Another has indicated that the logotherapy approach could be a useful, vital approach for reducing psychological disturbance in people who suffer from chronic physical illnesses (36). Other research in patients undergoing peritoneal dialysis and hemodialysis has aimed to investigate the personal abilities of self-distancing, self-transcendence, freedom, and responsibility in dialysis patients compared toa control group. The results have indicated that logotherapy caused improvements in daily activities among people who had dialysis treatment (37).

As with our results, evidence has confirmed the positive impact of other approaches, specific stress management intervention, and group therapy on the mental health of infertile couples and other important effects of these approaches on pregnancy rates (38).

Some studies have stated that group therapy and other approaches did not improve pregnancy rates, but rather they noted decreased rates of depression and anxiety (39).

Psychological interventions can be used as appropriate methods for infertile women who are not undergoing medical treatment (40). Stress management techniques should be offered to patients before, during, and after they undergo assisted conception treatments (41). Some studies agree with recent results that confirm the impact of group therapy in decreasing stress, depression, and anxiety in infertile women (42). Studies have shown that psychological intervention can often help in reducing stress, but they rarely increase the rate of pregnancy (41). The findings of the present study regarding the impact of logotherapy on reduce worry and perceived stress on infertile women (42).

## Conclusion

Logotherapy can be a sufficient method to reduce stress, worries, and other symptoms. This approach may of benefit for infertile couples' mental health. Thus, it can be concluded that logotherapy and attention to the spiritual aspect of patients who suffer from infertility may be a sufficient intervention during all stages of the infertility treatment. Further research is needed to understand whether other psychological approaches have the same consequences on infertility.
